# Following Excitation/Inhibition Ratio Homeostasis from Synapse to EEG in Monogenetic Neurodevelopmental Disorders

**DOI:** 10.3390/genes13020390

**Published:** 2022-02-21

**Authors:** Lisa Geertjens, Torben W. van Voorst, Arianne Bouman, Maaike A. van Boven, Tjitske Kleefstra, Matthijs Verhage, Klaus Linkenkaer-Hansen, Nael Nadif Kasri, L. Niels Cornelisse, Hilgo Bruining

**Affiliations:** 1 Child and Adolescent Psychiatry and Psychosocial Care, Emma Children’s Hospital, Amsterdam UMC, Vrije Universiteit Amsterdam, 1081 HV Amsterdam, The Netherlands; l.m.g.geertjens@amsterdamumc.nl; 2 N=You Neurodevelopmental Precision Center, Amsterdam Neuroscience, Amsterdam Reproduction and Development, Amsterdam UMC, Meibergdreef 5, 1105 AZ Amsterdam, The Netherlands; 3 Department of Functional Genomics Center for Neurogenomics and Cognitive Research (CNCR), VU University Amsterdam and Amsterdam UMC-Location VUmc, de Boelelaan 1085, 1081 HV Amsterdam, The Netherlands; t.w.van.voorst@vu.nl (T.W.v.V.); m.a.van.boven@vu.nl (M.A.v.B.); m.verhage@vu.nl (M.V.); l.n.cornelisse@vu.nl (L.N.C.); 4 Department of Human Genetics, Radboud University Medical Center, 6500 HB Nijmegen, The Netherlands; arianne.bouman@radboudumc.nl (A.B.); tjitske.kleefstra@radboudumc.nl (T.K.); n.nadif@donders.ru.nl (N.N.K.); 5 Department of Human Genetics, Center for Neurogenomics and Cognitive Research (CNCR), Amsterdam UMC-Location VUmc, de Boelelaan 1085, 1081 HV Amsterdam, The Netherlands; 6 Department of Integrative Neurophysiology, Center for Neurogenomics and Cognitive Research (CNCR), Amsterdam Neuroscience, VU University Amsterdam, 1081 HV Amsterdam, The Netherlands; k.linkenkaerhansen@vu.nl; 7 Levvel, Center for Child and Adolescent Psychiatry, Meibergdreef 5, 1105 AZ Amsterdam, The Netherlands

**Keywords:** neurodevelopmental disorders, iPSC-based models, EEG, SNAREopathies, chromatinopathies

## Abstract

Pharmacological options for neurodevelopmental disorders are limited to symptom suppressing agents that do not target underlying pathophysiological mechanisms. Studies on specific genetic disorders causing neurodevelopmental disorders have elucidated pathophysiological mechanisms to develop more rational treatments. Here, we present our concerted multi-level strategy ‘BRAINMODEL’, focusing on excitation/inhibition ratio homeostasis across different levels of neuroscientific interrogation. The aim is to develop personalized treatment strategies by linking iPSC-based models and novel EEG measurements to patient report outcome measures in individual patients. We focus our strategy on chromatin- and SNAREopathies as examples of severe genetic neurodevelopmental disorders with an unmet need for rational interventions.

## 1. Introduction

Neurodevelopmental disorders (NDDs) are highly heterogenous in etiology and manifestation, and cause tremendous suffering for patients and caregivers. Current treatments are limited to generic symptom suppressing medications that do not take heterogeneity into account. There is a need for mechanism-based therapeutic options to remedy the life-long suffering of patients and caregivers often associated with NDDs.

The identification of risk genes for NDDs provides new starting points for mechanism-based therapies. For example, in recent work, 102 risk genes for autism spectrum disorder (ASD) have been identified [1]. The discovery of NDD-associated de novo variants in genes with roles in synaptic plasticity has provided an entry to start developing rational interventions. Indeed, NDDs caused by variations in single genes, so called monogenetic NDDs (mNDDs), seem to converge on a disturbed balance between excitatory and inhibitory inputs (E/I) in neuronal networks in the brain [2,3,4,5,6,7,8] that occur early (1st/2nd trimester), or in early postnatal stages [1]. Although the concept is rather generic and applied in many contexts, it is well established that cortical networks require a finely tuned coordination of excitatory and inhibitory inputs for normal information processing [9], and that changes in both directions (increasing or decreasing E/I ratio) may compromise processing and lead to NDD clinical symptoms. The E/I-balance concept is further supported by NDD mouse model studies that show E/I ratio disturbances [10] and EEG abnormalities in NDD patients that suggest E/I ratio imbalances [11,12,13]. Finally, we have recently reported initial successes with off-label medication targeting E/I-regulation [14,15,16]. Thus, influencing E/I ratios is regarded as a promising target for pharmaceutical interventions, but is complicated by the multifaceted heterogeneity of underlying mechanisms and thus requires personalized treatment strategies [17].

**Figure 1:**
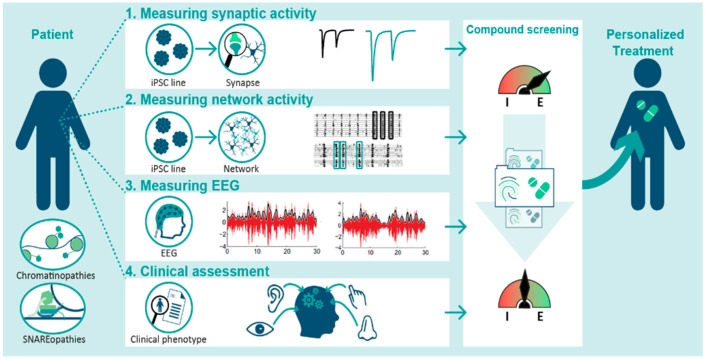
BRAINMODEL’s multi-level strategy.

Here, we first outline the heterogeneous nature and consequences of E/I ratio disturbances observed in NDD model research, which emphasizes the need for personalized treatment development strategies. We put forward that induced pluripotent stem-cell (iPSC)-based models provide new opportunities for translatability of E/I ratios to network activity homeostasis as proposed by BRAINMODEL. This project is conducted by a publicly funded Dutch consortium of neuroscientists and clinicians, and aims to develop personalized E/I targeting treatments through the linking iPSC-based models, EEG data, and clinical assessments in patients with two forms of genetic NDDS, chromatin-, and SNAREopathies (Figure 1).

## 2. Molecular and Physiological Heterogeneity of E/I Ratio Homeostasis in NDDs

Alterations in E/I ratio homeostasis in NDDs can result from aberrations in several processes, including synapse development, synaptic transmission, and neuronal excitability [18]. For example, synaptic E/I ratio changes have been described in human iPSC-derived neurons from Rett-Syndrome patients carrying *MECP2* loss-of-function mutations, resulting in decreased excitatory synaptic activity with no change in inhibitory activity [19]. Likewise, iPSC-derived neurons from individuals with Phelan–McDermid syndrome (PMDS) and autism showed selective defects in excitatory, but not inhibitory, synaptic transmission [20], and disruption of the autism-associated gene *SYNGAP1* increased excitatory synapse numbers in developing human neurons [21]. Alternatively, neurological phenotypes associated with E/I ratios changes have been observed as the broadening of action potentials in neurons derived from individuals with Timothy syndrome [22], while the genetic deletion of the Angelman Syndrome-associated gene *UBE3A* was shown to increase the excitability of induced human neurons [23]. Similarly, human neurons KO for the Fragile X Syndrome-associated gene *FMR1* exhibited increased intrinsic excitability, with no discernible synaptic phenotype [24]. In addition, altered synaptic E/I ratios and neuronal excitability phenotypes often co-occur. For instance, NDD-associated variants of the synaptic protein CASK appear to reduce the size of inhibitory presynaptic compartments, while simultaneously reducing spiking activity [25]. Likewise, the conditional deletion of the PMDS-associated gene *SHANK3* did not only produce hyperexcitability through modulation of intrinsic membrane properties, but also produced extensive synaptic impairments [26].

## 3. The Potential and Pitfalls of E/I Ratio Measurements in iPSC Models

The above findings underline the potential of models consisting of neurons and neuronal networks derived from *patient-own* tissues using iPSC-technology. These may bridge the gap between in vitro models and in vivo manifestations [27], and for instance, link different levels of E/I ratio homeostasis in response to treatment. The generation of glutamatergic and GABAergic neurons from patient-derived iPSCs can be achieved either using dual-SMAD inhibition [28] or through the ectopic expression of transcription factors [29,30,31], of which the latter is better suited to robust high-throughput assays in terms of both scalability, and cellular and maturational homogeneity [32,33,34]. The use of single-neuron (“autapse”) cultures [35,36] generated from induced glutamatergic and GABAergic neurons (iNeurons) grown in isolation on microdot arrays, allows for robust and standardized analysis of cell-autonomous synaptic functioning and intrinsic excitability without the interference of homeostatic mechanisms. Conversely, co-culturing patient-derived glutamatergic and GABAergic iNeurons on multi-electrode arrays (MEAs) allows for recording of network activity and analysis of network E/I ratio [37]. Moreover, by recording the development of neuronal network behavior over time, the interactions between the primary disfunctions in synaptic activity or excitability and maturation checkpoints can be studied, such as the development of mature intracellular chloride levels [31].

It is important to note that methodologies to develop human-neurons from IPSCs are still actively being developed and optimized, and it has become clear that neurons generated in vitro differ substantially from those found in the human brain [38]. Thus, one should be aware that while these cells resemble human neurons, they are not necessarily identical to those found in the human brain. Nevertheless, it is clear that induced neurons have a clear neuronal morphology, are electrophysiologically active, express markers for specific neuronal lineages also found in the human brain, and form interconnected networks able to integrate into the developing brain in mice in vivo [39,40], illustrating its validity as a model for (developing) human neurons. In addition, in the developing brain, neurons receive many inputs from many different cell-types, which are under strict spatiotemporal control. This cannot be accurately modeled in in vitro systems. Thus, readouts obtained from these systems should always be interpreted in its context as a simplified model.

Furthermore, these methodologies come paired with moral and ethical issues. As this novel strategy has yet to prove its efficacy as a tool for the selection of therapeutic interventions, patients and other stakeholders might be reluctant to participate in this study. Another concern is that cultured human neurons are often portrayed as ‘miniature brains’, suggesting that there is a possibility that consciousness could be generated in cultured neurons. This brings forward concerns regarding the moral status of said cultured neuronal networks. However, its highly improbable that cultured neurons in the methodologies currently employed could be conscious, as the number of neurons and the complexity of the networks in these systems are low, even compared to cultured neuronal organoids, which resemble the human cortex to a greater extent and for which this concern is more relevant [41,42].

## 4. Focus on Chromatinopathies and SNAREopathies

In the BRAINMODEL project, we will initially focus our multi-level phenotyping on two classes of mNDDs. We thereby aim to collect observations from multiple individuals with variants in the same gene to identify cellular and network hallmarks and targets for these disorders, as we have previously done for Kleefstra-syndrome and *STXBP1*-encephalopathy, part of the so-called chromatinopathies and SNAREopathies, respectively [43,44]. Indeed, using CRISPR/Cas9-technology, the genotype of the iPSCs can be altered, inducing disease-associated variants in healthy cells or repairing the variant in patient-derived cells [45], demonstrating causal relationships between detected phenotypes and patient genotype. After the identification of cellular and network deficits, therapeutic strategies to correct these aberrations can be selected and tested in vitro as performed by Marchetto et al. and Yahata et al. for Rett syndrome and Alzheimer’s disease, respectively [19,46]. In BRAINMODEL, we will expand our expertise on iPSC characterization of chromatinopathies and SNAREopathies and focus on treatment development for these two classes of mNDDs.

**Figure 2:**
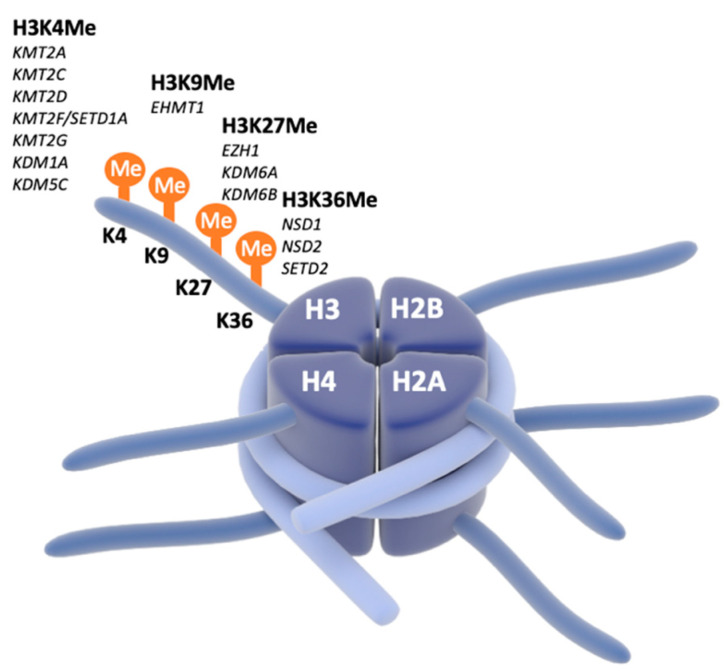
Transcription regulation is controlled by histone modifications. Schematic representation of the deposition of histone methylation, performed by histone methyltransferases and histone demethylases on histone 3 (H3), which have been linked to neurodevelopmental disorders.

Chromatinopathies are found to be major contributors to NDDs [1,47]. Chromatin remodeling determines whether genes are available for transcription and is crucial in active regulation of gene expression. We focus on four different monogenic disorders caused by a pathological mutation or deletion in genes (*EHMT1*, *KMT2C*, *KMT2D* and *SETD1A*) coding for enzymes which carry out chromatin remodeling (e.g., methylation) (Figure 2). Although mNDDs caused by mutations in these genes have the same neurobiological etiology (altered chromatin remodeling), there is a high variability in clinical presentation. Apart from intellectual disability (ID) and/or developmental delay (DD) presenting in almost 100% of cases, core symptoms are childhood hypotonia, psychiatric disorders (including autism-spectrum disorder (ASD), attention-deficit disorder (ADHD), and anxiety), epilepsy, and sleep disorders. In addition, facial dysmorphisms are present, and anomalies are found in several organ systems [48,49,50,51]. As for the E/I ratio homeostasis, previous studies showed that the Loss of function (LoF) of *EHMT1* results in delayed GABAergic maturation, reduced inhibition, and hence increased E/I ratio [52,53,54].

**Figure 3:**
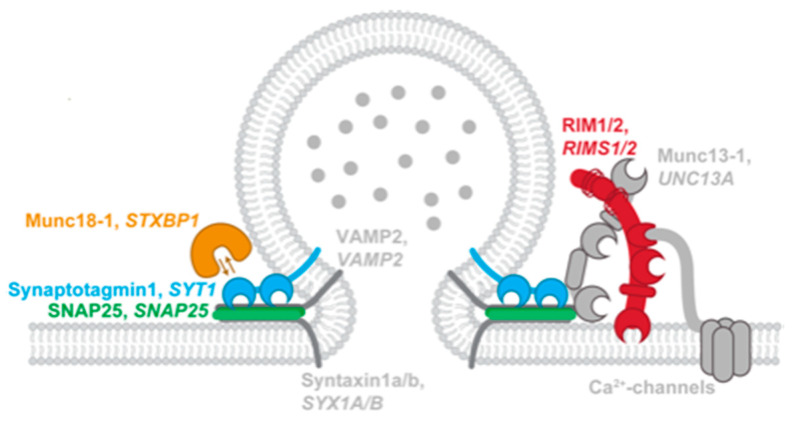
Schematic Representation of the eight SNAREopathy genes with their orientation relative to the synaptic vesicle and the plasma membrane and their interaction.

SNAREopathies are another group of pathobiological well-defined mNDDs. These disorders, caused by mutations that disturb SNARE function, are a subset of the previously defined synaptopathies. The neuronal SNARE complex (soluble NSF attachment protein receptor complex) is an important molecular machine driving synaptic vesicle exocytosis and secretion of neuropeptides and neuromodulators from dense core vesicles [55]. We focus on four of these genes that are associated with NDDs (*STXBP1, SYT1, SNAP25, RIMS1*) (Figure 3). Although the pathogenic starting point of these disorders is well defined, clinical phenotype and disease severity is very diverse. Moreover, high clinical variety is found in the same amino acid changes between different individuals [55]. Most common clinical aspects found in SNAREopathies are ID and/or DD, seizures, ASD, and neurological motor problems. Even though almost all cases present with ID, the mechanisms through which mutations in SNARE genes lead to neurodevelopmental impairments remain unexplained. Additional genetic and/or environmental factors might contribute substantially to disease presentation and should be considered when studying disease mechanisms [55]. Based on mouse models, mutations in SNAREopathy genes also create a disturbed E/I ratio setpoints [55]. However, it is unknown to which extent the different components in the E/I microcircuits in the brain are susceptible to gene mutations [55].

## 5. Connecting the Dots

There is need for caution in interpreting neuronal and network phenotypes, as an apparent E/I phenotype might be the result of homeostatic compensation mechanisms [6] rather than a cell-autonomous NDD phenotype [56]. This is illustrated by the finding that the pharmacological induction of hyperexcitability was sufficient to phenocopy *SHANK*^−/−^-associated synaptic defects in wild type neurons [26], similar to findings in mouse models, where an altered E/I ratio was found to be a compensatory mechanism to stabilize the circuit [10]. Indeed, the applicability for therapeutic screening in IPSC based models is limited, as the model does not represent a full organism. Pharmacodynamics and -kinetics are different, for example, due to the incapacity to model the blood brain barrier. Thus, whether a potential therapeutic intervention can reach the target cells in vivo cannot be determined in these models. Furthermore, off-target effects at other areas of the body cannot be studied. iPSC-based models do, however, provide the opportunity to test novel compounds in vitro in advance of clinical trials. This enables the identification of more therapeutic options, either based upon existing or new compounds, where the former has the advantage of knowledge on pharmacodynamics and -kinetics.

**Figure 4:**
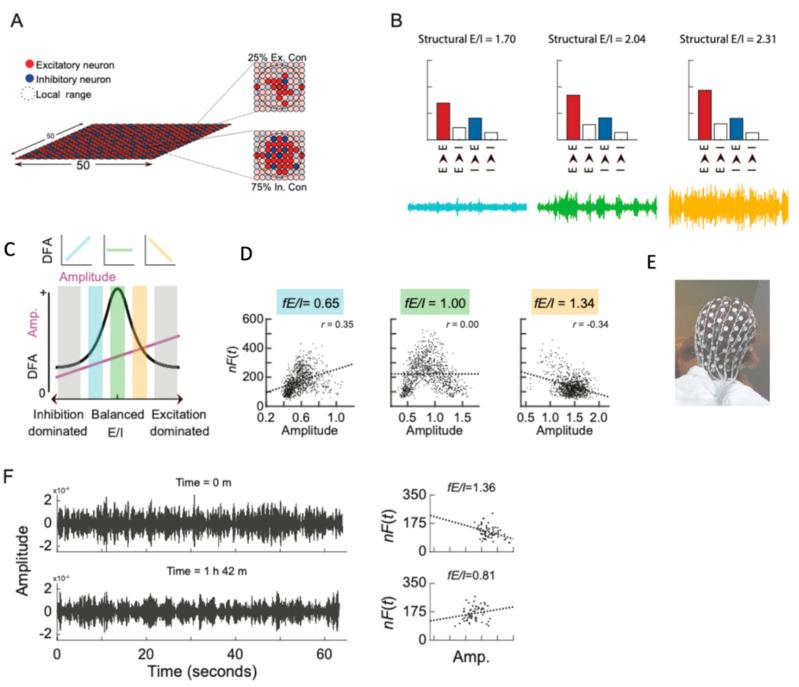
E/I estimation—from model to human EEG measurements: (**A**) The critical oscillations model simulates excitatory (red) and inhibitory (blue) neurons situated in a network. The E/I ratio can be regulated by changing the percentages of excitatory and inhibitory neurons that a neuron connects to within a local range (dashed lines). (**B**) Increasing excitatory connectivity in model networks (red bars, top row) leads to increasing amplitude of oscillations (bottom row). (**C**) The amplitude of oscillations (purple line) increases with increasing excitation, whereas the temporal complexity as quantified by the detrended fluctuation analysis (DFA, black line) peaks when excitation and inhibition is balanced. This relationship implies that a windowed analysis of oscillations reveals either positive, zero, or negative correlations (top inserts). (**D**) Hence, we defined a biomarker of E/I ratios as 1 minus the correlation, r, between windowed power and DFA (E/I = 1 – r), and showed that the structural E/I is well estimated by the E/I biomarker (fE/I) in simulated oscillations in networks with different structural E/I ratios. (**E**) Thus measuring EEG and (**F**) performing a joint analysis of the power and temporal structure of oscillations allows estimating individual differences in cortical E/I ratios or how these are pharmacologically modulated (Adjusted summary Figure of Bruining et al., 2020) [12].

To complement the multi-level strategy on a neurophysiological level, resting state electroencephalography (rsEEG) recordings can be analyzed in the same patients. We have put forward that the concept of critical brain dynamics is a steppingstone to derive E/I ratios from neuronal oscillations measured with conventional EEG [57,58,59,60] (represented in Figure 4B) [58,61]. Our computational modeling [60] (Figure 4A), as well as pharmacological challenges [61], have indicated that the so-called ‘critical’ regime between low and high activity requires balance between excitation and inhibition. Therefore, the basis of the E/I method is the statistical character of activity in this critical state where long-range temporal correlations (LRTC) [60] weaken when network E/I is out of balance [61,62] (Figure 4C,D). Therefore, we could use LRTCs to estimate E/I ratios leading to a functional E/I measure (fE/I) (Figure 4E–G). We validated this fE/I method at rest (rsEEG) and after GABAergic treatment. In children with ASD, we corroborated that both increased and decreased E/I ratios may contribute to ASD [12]. In BRAINMODEL, we will perform rsEEGs to evaluate these markers. In addition, we will perform source localization analyses to evaluate the importance of specific markers in specific brain areas. The use of this multi-level strategy provides the opportunity to evaluate direct consequences of mutation in patient-derived IPSCs and long-term (possibly compensatory) mechanisms in the network (EEG).

Finally, we developed a set of Patient Reported Outcome Measures (PROMs) that will be used for clinical endpoint measurement in BRAINMODEL [63]. Indeed, most existing clinical NDD measures focus on core symptom definition and have been developed for diagnostic characterization. They have limited utility as read-outs of specific mechanistic perturbations and are psychometrically often not suitable as outcome measures for intervention studies [64,65]. To overcome this, we have recently investigated how sensory reactivity problems, recently added as a core domain element for ASD in the DSM, may extend into problematic behavior or affective dysregulation and how disturbed E/I ratio homeostasis may be translated into clinical readout measures [63]. This resulted in the PROM for the repeated and reliable measurement of patient-relevant consequences of sensory reactivity alterations [63] that we developed by following the FDA steps for (parent proxy) PROM for clinical trials [66,67]. According to this protocol, we initiated focus groups and interviews with caregivers to elicit the most impactful and most relevant symptoms and then sought to measure these PROs with large item banks of the Patient-Reported Outcomes Measurement Information System^®^ (PROMIS) [68], initiated by the “NIH Roadmap Initiative”, based on Item Response Theory (IRT) with the possibility to use Computerized Adaptive Testing (CAT) [69].

## 6. Conclusions

Previous research has provided crucial starting points to understand pathophysiological mechanisms in NDDs to develop therapeutic options. IPSC-based models for mNDDs have unprecedented promise to bridge the gap between well-established animal and cellular models towards human treatment development. In BRAINMODEL, we will employ a multi-level strategy in which iPSC based-models, neurophysiological parameters, and PROMs are combined within the framework of chromatin- and SNAREopathies in order to develop personalized mechanism-based therapeutic strategies targeting E/I ratio homeostasis. 

## Data Availability

Not applicable.
